# Resolving RAD51C function in late stages of homologous recombination

**DOI:** 10.1186/1747-1028-2-15

**Published:** 2007-06-04

**Authors:** Shyam K Sharan, Sergey G Kuznetsov

**Affiliations:** 1 Mouse Cancer Genetics Program, Center for Cancer Research, National Cancer Institute, Frederick, Maryland 21702, USA

## Abstract

DNA double strand breaks are efficiently repaired by homologous recombination. One of the last steps of this process is resolution of Holliday junctions that are formed at the sites of genetic exchange between homologous DNA. Although various resolvases with Holliday junctions processing activity have been identified in bacteriophages, bacteria and archaebacteria, eukaryotic resolvases have been elusive. Recent biochemical evidence has revealed that RAD51C and XRCC3, members of the RAD51-like protein family, are involved in Holliday junction resolution in mammalian cells. However, purified recombinant RAD51C and XRCC3 proteins have not shown any Holliday junction resolution activity. In addition, these proteins did not reveal the presence of a nuclease domain, which raises doubts about their ability to function as a resolvase. Furthermore, oocytes from infertile *Rad51C *mutant mice exhibit precocious separation of sister chromatids at metaphase II, a phenotype that reflects a defect in sister chromatid cohesion, not a lack of Holliday junction resolution. Here we discuss a model to explain how a Holliday junction resolution defect can lead to sister chromatid separation in mouse oocytes. We also describe other recent *in vitro *and *in vivo *evidence supporting a late role for RAD51C in homologous recombination in mammalian cells, which is likely to be resolution of the Holliday junction.

## Background

**Figure 1:**
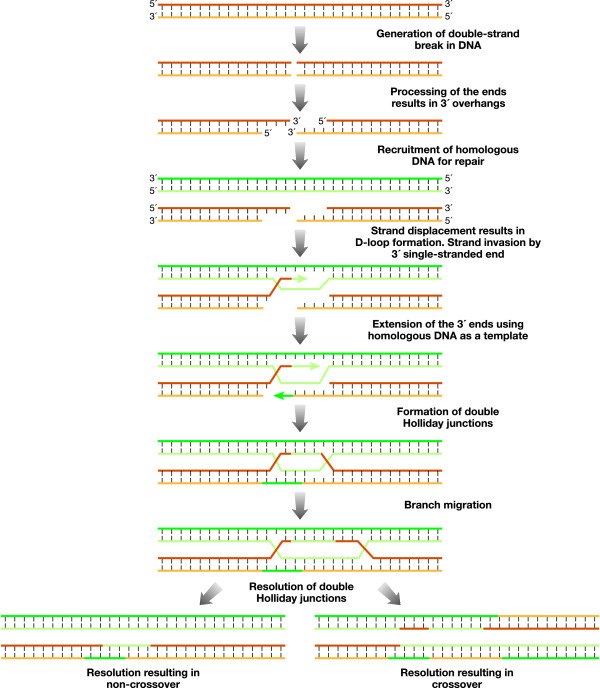
**Schematic representation of the model showing repair of DSBs by homologous recombination**. When a DSB occurs, it is processed to generate 3' single-stranded ends. RAD51 nucleoprotein microfilament is assembled on these ends, one of which invades the homologous DNA by strand displacement. This results in the formation of the D-loop. The invading strand primes DNA synthesis using the homologous DNA as a template. The second single-stranded DNA is also captured for priming DNA synthesis by using the displaced strand as a template. Extension of the 3' ends ultimately results in two cross-structures that hold the two homologs together, called Holliday junctions (HJs). Migration of HJs, called branch migration, results in the formation of heteroduplex regions. The double HJs are resolved by resolvases and, depending on the cleavage site, can either lead to crossover or non-crossover products. Resolution to yield crossover products requires symmetric cleavage of both HJs in opposite orientations.

Exchange of genetic material by homologous recombination during meiosis is essential for generating genetic diversity in living organisms. It also results in the formation of chiasmata, that are the cytological manifestation of crossovers and ensure proper chromosome segregation. Homologous recombination is important for error-free repair of damaged DNA as well [1]. One of the key steps of this process is the exchange of DNA strands between homologous chromosomes, which results in the formation of a cross-stranded DNA structure (Figure 1). More than 40 years ago, Robin Holliday proposed the formation of such an intermediate during DNA recombination [2]. It was hypothesized that such recombination intermediates, now known as Holliday junctions (HJs), can be resolved by the endonucleases capable of binding to such specialized DNA structures [3]. In 1982, the first resolvase was identified [4]. It was the bacteriophage T4 endonuclease VII, which was shown to cleave Holliday structures *in vitro *by nicking two strands of the same polarity near the branch point. The breaks generated as a result of this cleavage could be sealed by a DNA ligase, resulting in intact resolved products. A search for resolvases in other organisms resulted in the identification of one of the best-characterized resolvases, RuvC of *E. coli *[5,6], which resolves Holliday junctions by introducing concerted nicks across the junction branch point. Two additional proteins, RuvA and RuvB, have been shown to be an integral part of the resolvosome complex that facilitates the movement of HJ along the DNA in an ATP-dependent manner, in a process known as branch migration (Figure 1) [7]. Recently, resolvases that share some structural similarity with *E. coli *RuvC have been identified like CCE1 in yeast, RuvC in *Lactoccocal *bacteriophage and A22R in Poxvirus [8-11]. Some other distinct resolvases have been identified in bacteriophages, such as the T7 endonuclease I, RusA and Rap [12,13]. In addition, Hjc and Hje proteins from archaebacteria demonstrate HJ cleaving activity [14-16].

### Eukaryotic resolvases

Although many resolvases have been identified in prokaryotes, few have been found in eukaryotes. The yeast CCE1 is encoded by a nuclear gene but functions as a resolvase in the mitochondria [9,17]. Identification of the Mus81-Eme1 endonuclease in *Saccharomyces pombe *raised hopes that the eukaryotic resolvase has been identified [18]. It has a substrate preference for nicked HJs and displacement loops (D-loops) [19]. Genetic studies showed that *S. pombe mus81 *mutants are infertile due to failed meiotic recombination, which could be rescued by the expression of bacterial RusA resolvase [18]. These studies demonstrated that Mus81 is indeed a eukaryotic resolvase. The homologs of fission yeast Mus81-Eme1 have been identified in other organisms, including *Saccharomyces cereviciae *(Mus81-Mms4), *Arabidopsis *(AtMUS81/At4g30870) and humans (Mus81-Eme1 or Mms4) [20-26]. However, unlike the *S. pombe *mutants, *S. cereviciae mus81 *mutants are partially fertile, and MUS81-deficient *Arabidopsis *plants and mice are fully fertile, with no defect in meiotic recombination [21-23,27,28]. These observations suggest the presence of other resolvases in these organisms. Also, it was shown that the HJ resolution activity in mammalian cells could be separated from MUS81, suggesting that MUS81 was not the mammalian HJ resolvase [29].

### RAD51 paralogs

The idea that RAD51 paralogs may be involved in resolution of HJs came from the observation that the protein fraction with HJ resolution activity contained RAD51C and XRCC3, members of the RAD51-like protein family [30]. This observation was very exciting considering that members of this family were already known to play a role during the early stages of homologous recombination. In higher eukaryotes, including plants, chicken and mammals, there are six members of the RAD51-like protein family that show 20–30% sequence similarity to RAD51 [31,32]. These are RAD51B/RAD51L1, RAD51C/RAD51L2, RAD51D/RAD51L3, XRCC2, XRCC3 and DMC1. DMC1 shares about 50% sequence identity with RAD51and is its structural and functional homolog that functions specifically in meiotic recombination [33]. The other five paralogs show 20–30% sequence similarity to RAD51 and have been shown to be part of at least two distinct protein complexes, namely, the BCDX2 and CX3 complexes [34]. The BCDX2 complex comprises RAD51B, RAD51C, RAD51D and XRCC2 proteins; the CX3 complex consists of RAD51C and XRCC3. All these RAD51 paralogs have been reported to be required for normal proliferation and play a role in RAD51-mediated homologous recombination [31].

Studies in chicken B-lymphocyte DT40 cells have shown that only RAD51 is essential for cell viability, while loss of other RAD51 paralogs does not affect cell survival [35]. Interestingly, although mutants lacking any of the RAD51 paralogs show sensitivity to DNA-damaging agents, their phenotypes are not identical, suggesting that their function is similar but not redundant [36]. This functional non-redundancy is corroborated by the loss-of-function studies in mice and *Arabidopsis *[37-40]. Depletion of RAD51C by siRNA in human cells also suggests that RAD51C plays a role in homologous recombinational repair [41]. These findings are consistent with the idea that these paralogs play a role in early stages of homologous recombination. Recent work from Stephen West's laboratory has suggested that RAD51C and XRCC3 may also play a role in the late stages of homologous recombination [30,42]. Here we discuss various findings that support the dual role of RAD51C.

### The role of RAD51C and XRCC3 in HJ resolution

In a very significant study, Liu et al. demonstrated that RAD51C is required for the resolution of HJs in mammalian cells [30]. After an intricate series of HeLa cell nuclear extract fractionations, fractions with HJ resolution and branch migration activity were identified. These fractions lacked some of the possible candidates like Mus81, Flap endonuclease 1 (FEN1), RecQ DNA helicase (BLM) and Werner syndrome helicase (WRN). On the other hand, all the fractions with HJ resolution activity contained RAD51C. Immunodepletion using a RAD51C antibody resulted in the loss of the HJ processing function. This activity could be restored by adding purified RAD51 paralog protein complexes containing RAD51C, but not those lacking it. These studies directly implicated RAD51C in the resolution process. Furthermore, the Chinese hamster ovary cell line *irs3 *defective in RAD51C was shown to lack the HJ processing function. Recently, mouse embryonic fibroblasts generated from *Rad51c *knockout embryos revealed marked reduction in HJ activity [43]. Interestingly, a hamster cell line with mutant *XRCC3*, *irs1SF*, also showed a defect in HJ resolution activity, while mutation in another RAD51 paralog, XRCC2, had no effect on HJ resolution [30]. Although Liu et al. provided strong evidence to show the involvement of both RAD51C as well as XRCC3 in the resolution of HJ, a direct cleavage of the HJ structure by purified recombinant proteins remains to be demonstrated. It is possible that these proteins undergo posttranslational modifications, which may be essential for the HJ processing function. This theory is supported by the observation that the mobility of the RAD51C present in the fractions of HeLa cell extract that possess HJ resolution function is different from the mobility of those present in unfractionated whole cell extracts. Identifying the nature of this modification may elucidate the structural or conformational change in RAD51C that may be essential to its HJ resolution activity.

More recently, in a follow-up study, Liu et al. provide new biochemical evidence to link RAD51C and XRCC3 to the HJ processing function [42]. Using affinity chromatography, they showed a direct link between RAD51C and HJ activity. In a nickel column loaded with recombinant His-tagged RAD51C protein, HJ activity present in HeLa cell extracts was shown to bind to the column, which could subsequently be eluted. XRCC3 present in the extract was also shown to bind to the column and elute with HJ activity. Using gel filtration, the HJ resolvase activity was found to elute with an average molecular mass of 80–90 kDa, similar to the sum of the molecular masses of RAD51C (~42 kDa) and XRCC3 (~38 kDa). This finding suggests that the RAD51C-XRCC3 heterodimer should have the HJ activity. Because efforts to demonstrate this activity using purified recombinant proteins have failed, it has been speculated that the resolvosome complex may contain an additional component, such as a small nuclease, which may be essential for the HJ resolution function of the RAD51C-XRCC3 dimer. This suggestion may also explain the absence of any apparent nuclease domain in RAD51C or XRCC3. Future studies aimed at determining whether the HJ resolvosome complex involves other component(s) in addition to RAD51C and XRCC3 are important.

### Localization of RAD51C on meiotic chromosomes

In addition to the biochemical studies, Liu et al. have presented interesting immunofluorescence data showing the localization of RAD51C on meiotic chromosomes, which further supports its role in late stages of homologous recombination [42]. Since various stages of meiotic prophase I coincide well with different stages of double-strand-break (DSB) repair by homologous recombination, it is possible to associate the function of a protein based on its localization on meiotic chromosomes at any given prophase I stage. Interestingly, RAD51C was first detected at the pachytene stage, when it was observed as one or two distinct foci associated with each synapsed bivalent. This pattern of expression is similar to the foci formed by the mismatch repair protein MLH1 [44]. These foci are believed to represent the sites of crossovers, formed as a result of recombination between homologous chromosomes. In addition, spermatocytes from *Mlh1*-deficient mice, which have a severely reduced number of crossover sites, also showed a marked reduction in RAD51C foci. This observation strengthens the notion that RAD51C foci formation during pachytene depends on the generation of crossovers, which is one of the last steps of homologous recombination. Surprisingly, co-localization of MLH1 and RAD51C foci on the bivalents was not observed. Also, XRCC3 foci were not detected on these bivalents. These observations raise concerns and are currently difficult to explain. The lack of co-localization of RAD51C and MLH1 may be due to temporal differences in their localization at crossover sites. It is interesting that, although no XRCC3 foci were observed on autosomal bivalents, XRCC3 foci, along with RAD51C and MLH1 foci, were present in the pseudoautosomal region of the sex chromosomes, a region on X and Y chromosomes that undergoes an obligatory crossover event. These foci in the pseudoautosomal region were not detected in *Mlh1*-deficient spermatocytes, supporting their dependence on a crossover event.

It was surprising that Liu et al. did not detect RAD51C foci during the leptotene and zygotene stages of prophase I, where it is expected to play an important role based on its known function in the early stages of homologous recombination. However, failure to observe RAD51C foci does not rule out its functional importance at this stage. Different antibodies and more sensitive imaging methods may help resolve this discrepancy. In the meantime, it may be interesting to examine the consequence of the loss of these proteins in an *in vivo *model system. In *Drosophila*, the loss of RAD51C-like protein encoded by *spn-D *gene results in a meiosis-specific defect and may play a role similar to DMC1 [45]. Recent studies in *Arabidopsis *revealed that RAD51C is essential for repair of Spo11-induced DSBs during prophase I of meiosis [40,46]. Homologous chromosomes in *Rad51c*-deficient plant meiocytes fail to synapse and become severely fragmented. These results support a role for RAD51C early in meiotic recombination but do not shed light on its role later in the process.

### RAD51C function in meiosis

Because RAD51C is predicted to have a dual function, an early and a late role in homologous recombination, it may be difficult to demonstrate the latter function *in vivo*, as a defect in the former may result in the arrest of meiocytes. Because RAD51C-deficient mice die during embryogenesis and no suitable meiosis-specific *Cre *transgenic mouse line is available yet, it is a challenge to generate a suitable mouse model to study the meiotic functions of RAD51C. We recently reported the generation of a hypomorphic allele of *Rad51c *that results in a reduction of the protein level due to aberrant splicing [43]. This aberrant splicing is caused by the presence of the neomycin resistance gene in one of the introns. Mice that are homozygous for the hypomorphic allele and have about 60% reduction in RAD51C protein level are viable and fertile. However, mice with only one copy of the hypomorphic allele (while the other allele is a null) are viable, but 35% of males and 12% of females are infertile. The infertile males and females provide an ideal *in vivo *model system to study the role of RAD51C in meiotic recombination. In addition, the meiotic phenotype associated with the loss of RAD51C function is sexually dimorphic, showing an early meiotic defect in males and a late defect in females, which provides a unique opportunity to study the dual function of RAD51C in mouse meiosis.

Infertile males revealed an early role for RAD51C in meiosis, marked by the spermatocyte arrest at leptotene and early zygotene stages, reduction of RAD51 foci at leptotene, and persistence of DNA breaks and unsynapsed chromosomes at pachytene. This finding is consistent with the known function of RAD51C in DSB repair but does not provide any evidence to implicate RAD51C in the late steps of the recombination.

**Figure 2:**
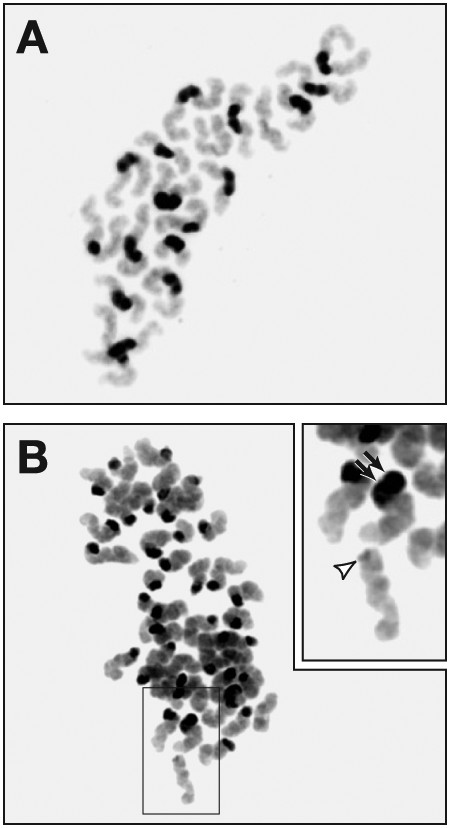
**A late role for RAD51C in meiotic recombination**. RAD51C's late role in meiotic recombination is revealed in oocytes that were allowed to progress to metaphase II *in vivo *following hormonal treatment. A. At metaphase II, oocytes from control females show the presence of 20 pairs of chromatids, each consisting of two sister chromatids that are attached at their centromeres. B. Oocytes from infertile *Rad51c *mutant mice display a variety of chromosomal abnormalities. The majority of the mutant oocytes show precocious separation of sister chromatids. Inset in "D" is a higher magnification of the group of chromosomes showing an acentric chromatid (arrowhead) and a chromatid with two centromeres (double arrow). (Reproduced from *The Journal of Cell Biology*, 2007, 176:581–592, Copyright 2007, The Rockefeller University Press.)

Histological examination of the ovaries of mutant females revealed a defect in ovulation, which could be partially rectified by hormonal treatment. The number of oocytes was markedly reduced, and the embryos that developed from these oocytes suffered from severe developmental defects. The reduced number of oocytes suggests that some oocytes may have been lost due to a defect in the early meiotic prophase I. While all oocytes obtained after superovulation progressed normally to metaphase I, they displayed a number of abnormal features at metaphase II. These abnormalities included aneuploidy and broken chromosomes, but most strikingly, precocious separation of sister chromatids (PSSC; Figure 2). The observation that the oocytes showed a defect after metaphase I supports the notion that RAD51C plays a role during late stages of meiotic recombination. Oocytes with a defect early in the DSB repair process show an aberrant phenotype at metaphase I, which is characterized by the presence of achiasmatic chromosomes [47]. However, the possibility that a defect in DSB repair can lead to a PSSC phenotype in oocytes cannot be ruled out. Mice lacking *Brca2 *in the gonads have been generated and shown to have a defect in the early stages of meiotic recombination [48]. *Brca2 *mutant oocytes displayed a wide range of abnormalities, including a 2.5-fold reduction in the number of oocytes that progressed to metaphase II, compared to controls and major defects in chromosome segregation into polar bodies. It will be interesting to determine whether these oocytes display any PSSC defect at metaphase II.

### How does an HJ resolution defect result in sister chromatid separation?

The broken chromosomes and aneuploidy observed in *Rad51c *mutant oocytes are consistent with RAD51C playing a role in HJ resolution. One can expect chromosomes to break if the bivalents do not separate in the absence of HJ resolution. Similarly, it is possible that bivalents can go to the same pole if they remain attached. However, in the *Rad51c *mutant oocytes, these phenotypes were not frequently observed. The most predominant phenotype displayed by the majority of chromosomes was PSSC, a typical characteristic of a defect in sister chromatid cohesion [49]. So, how can an HJ resolution defect result in this phenotype? Coincidently, a Chinese hamster ovary cell line lacking RAD51C was also reported to exhibit a PSSC defect [50]. Together, these findings raised the question of whether RAD51C may indeed be involved in sister chromatid cohesion. Investigation into a possible role for RAD51C as a cohesin by testing its physical interaction with known cohesins, examining the effect of the loss of RAD51C on the localization of other cohesins on meiotic chromosomes, and electron-microscopic analysis of synapsed chromosomes in mutant spermatocytes yielded no supporting data (Kuznetsov and Sharan, unpublished observation).

**Figure 3:**
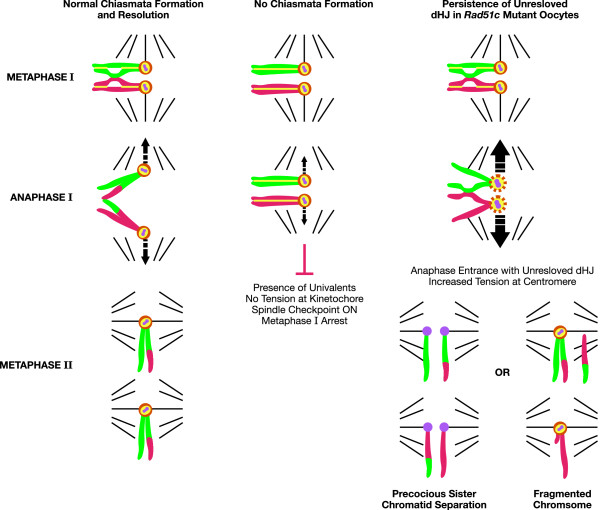
**Proposed connection between HJ resolution defect and abnormalities in *Rad51c *mutant oocytes at metaphase II**. During meiosis, HJs are established between homologous chromosomes by the pachytene stage of prophase I using the homologous recombination machinery. At metaphase I, bivalents are pulled in opposite directions by microtubules attached to kinetochores of sister chromatids that are oriented toward the same pole. While centromeric cohesion is protected by Shugoshin to ensure that sister chromatids stay together during the reductional division, cohesion is released along the chromosome arms. During anaphase I, homologous chromosomes segregate to separate cells. In the absence of chiasmata, homologous chromosomes do not align properly at the metaphase plate, and this activates a spindle checkpoint resulting in metaphase I arrest. In *Rad51c*-deficient oocytes, meiosis progresses normally until anaphase I. However, due to accumulation of recombination intermediates, such as double Holliday junctions (dHJs), which hold the homologous chromosomes together, there is an increase in tension at the centromere due to the persistence of unresolved dHJs. The increased tension is thought to disrupt the sister chromatid cohesion at the centromere, resulting in the PSSC phenotype and fragmented chromosomes. Homologous chromosomes are shown in red and green; REC8 is shown in yellow; shugoshin is orange; and centromeres are shown in purple. (Reproduced from *The Journal of Cell Biology*, 2007, 176:581–592, Copyright 2007 The Rockefeller University Press.)

Next, the option that a defect in sister chromatid cohesion could be the consequence of unresolved HJs persisting on meiotic chromosomes during anaphase I was explored [43]. It was proposed that the increased physical tension on sister centromeres at the onset of anaphase I may somehow disrupt their cohesion (Figure 3). This physical tension may arise if the homologs are unable to separate from each other when the HJs are not resolved. The homologs normally align at the metaphase plate, and the kinetochores are attached to the spindle, allowing them to be pulled to the opposite poles. This action ensures proper segregation of each homolog to one of the daughter cells at the end of meiosis I. Sister chromatid cohesion is established during the S-phase and lost between the sister chromatid arms prior to metaphase I [51]. However, the sister chromatids do not dissociate as they remain attached by their centromeres. The centromeric cohesion is protected from degradation by a conserved family of centromere-associated protein called shugoshin (Sgo) [52-54]. In meiosis, during anaphase II, when the sister chromatids separate from each other and move to opposite poles, shugoshin is degraded, exposing the centromeric cohesion complex to separase, leading to a loss of centromeric cohesion. Recent studies have shown that, in addition to the main function of protecting centromeric cohesion, shugoshin is involved in sensing tension at the kinetochore [55,56]. This tension-sensing mechanism is essential for activation of the spindle checkpoint. It has been demonstrated that a lack of tension between achiasmatic univalents activates the spindle checkpoint [57]. It is postulated that if the bivalents remain attached in *Rad51c *mutant oocytes due to unresolved HJs, an increase in tension is likely to occur at the kinetochore, which then may either physically disrupt the cohesion complex or may somehow activate the degradation of shugoshin, exposing the centromeric cohesion complex to separase (Figure 3).

Although these possibilities remain to be tested, a genetic evidence reported earlier was used to support this hypothesis [43]. Koehler et al. have shown that, in mouse oocytes, segregation of dicentric chromosomes very frequently (>90%) results in PSSC [58]. They proposed that the physical strain exerted on the homologous centromeres of the dicentric chromatid by the poleward microtubules can result in the PSSC cohesion. Kuznetsov et al. have suggested that unresolved chromosomes and dicentric chromosomes are likely to experience a similar mechanical stress at the centromere during anaphase I and therefore have a similar fate [43]. The processing of dicentric chromosomes is sexually dimorphic and so far has been reported only in mouse and human oocytes. In other organisms, such as maize and flies, such dicentric chromosomes are known to undergo a "bridge-fusion-breakage" cycle [59,60]. Why dicentric chromosomes have a different fate in mice and human oocytes is currently not understood. Interestingly, some of the spermatocytes from *Rad51c *mutant infertile males that progressed to metaphase II exhibited chromosomes with broken centromeres, but none showed any sister chromatid cohesion defect, which is consistent with the sexually dimorphic behavior of dicentric chromosomes.

In general, resolution of recombination intermediates in meiosis appears to be tightly linked to sister chromatid cohesion. The condensin-dependent removal of cohesin from the chromosome arms is required for efficient homolog separation in meiosis [61]. At the same time, the condensin – polo-like kinase axis is dispensable for cohesin removal in mitosis [62]. It is not clear how RAD51C might be involved in this particular process and whether it could explain the PSSC phenotype of the RAD51C-deficient mouse oocytes. However, it is intriguing that cohesins and RAD51C are now associated with the resolution of recombination intermediates after previously being independently implicated in the homologous recombination process [63].

## Conclusion

Three years since the initial report showing a role for RAD51C in HJ resolution in mammalian cells, are we any closer to resolving the biological function of RAD51C? In spite of all the biochemical experiments and examination of loss-of-function mutations in *Drosophila, Arabidopsis*, and mice, the late function in HJ resolution remains to be unequivocally demonstrated. The model to explain the phenotype of *Rad51c *mutant oocytes at metaphase II is intriguing but needs to be validated. It will be fascinating to directly observe the oocytes undergoing in vitro maturation by time lapse imaging to visualize the bivalents being pulled to opposite poles but remaining attached at the site of the crossover by chiasmata-like structures during anaphase I. Also, it will be interesting to examine the fate of shugoshin and cohesins on the centromeres that have undergone precocious separation. An alternative approach may be to bypass the early meiotic arrest during male meiosis by using a conditional *Rad51c *allele and generating appropriate meiosis-specific *Cre *transgenic lines. This approach may provide a more convincing phenotype and help explain the late role of RAD51C in homologous recombination. Similar studies on XRCC3 in meiotic recombination may also provide valuable clues.

## Competing interests

The author(s) declare that they have no competing interests.
